# A Genosensor Based on the Modification of a Microcantilever: A Review

**DOI:** 10.3390/mi14020427

**Published:** 2023-02-10

**Authors:** He Zhang, Shuang Yang, Jian Zeng, Xin Li, Rongyan Chuai

**Affiliations:** School of Information Science and Engineering, Shenyang University of Technology, Shenyang 110870, China

**Keywords:** microcantilever, genetic probe, sensitive modification, detection principle, environmental impact factors, application field

## Abstract

When the free end of a microcantilever is modified by a genetic probe, this sensor can be used for a wider range of applications, such as for chemical analysis, biological testing, pharmaceutical screening, and environmental monitoring. In this paper, to clarify the preparation and detection process of a microcantilever sensor with genetic probe modification, the core procedures, such as probe immobilization, complementary hybridization, and signal extraction and processing, are combined and compared. Then, to reveal the microcantilever’s detection mechanism and analysis, the influencing factors of testing results, the theoretical research, including the deflection principle, the establishment and verification of a detection model, as well as environmental influencing factors are summarized. Next, to demonstrate the application results of the genetic-probe-modified sensors, based on the classification of detection targets, the application status of other substances except nucleic acid, virus, bacteria and cells is not introduced. Finally, by enumerating the application results of a genetic-probe-modified microcantilever combined with a microfluidic chip, the future development direction of this technology is surveyed. It is hoped that this review will contribute to the future design of a genetic-probe-modified microcantilever, with further exploration of the sensitive mechanism, optimization of the design and processing methods, expansion of the application fields, and promotion of practical application.

## 1. Introduction

As a basic microelectromechanical system (MEMS) device, the microcantilever was researched as early as the 1960s [1,2]. With the invention and development of the atomic force microscope (AFM) in the 1990s [3], microcantilevers have gradually become a hotspot of MEMS research over the past two decades. When used for measuring force or mass changes, the microcantilever is not only a sensitive component that can perceive directly but also a conversion component that can transfer measurement results into mechanical deformation [4,5]. When used in biological, medical, or chemical applications, the microcantilever becomes a single-function conversion component. The modification layer coated on the free end of a microcantilever acts as a sensitive component [6]. The modification layer can react with or adsorb the samples and generate a corresponding original signal. The modification layer is an important element that expands the microcantilever’s function and determines the sensor linearity, response time, reproducibility, and service life.

In 1995, Thundat et al., evaporated a golden film on the surface of a silicon nitride cantilever for the first time [7,8]. They studied the resonance frequency regularity of a microcantilever by using the specific adsorption between golden film and mercury vapor, while obtaining the quantitative relationship between them. Since the early days of microcantilever sensitive modification technology, many scholars have used sputtering, coating, grafting, molecular self-assembly, and the other methods to modify the microcantilever in order to further expand the sensor’s application range, explore the detection limit, and improve detection resolution [9,10].

Among these modification techniques, due to the high sensitivity, accuracy, and fast response, the genetic probe has gradually become an important choice for the sensitive modification of a microcantilever sensor. After genetic probe modification, the microcantilever sensor can convert the molecular recognition signal into nanoscale mechanical displacement, which can realize high-resolution, high-throughput, and real-time detection without a label. In many research fields, such as target DNA sequence and protein molecule analysis, bacterial and virus identification, and compound and heavy metal ion detection, traditional detection cannot be achieved or has a poor effect, but the genetic-probe-modified microcantilever sensor achieves fruitful results [11,12]. In this paper, the main genetic probe modification steps for a microcantilever, including probe immobilization, complementary hybridization, signal extraction, and signal processing, are introduced in detail. Then, the detection principle of a microcantilever that uses a genetic probe as a sensing element, the environmental impact factors of the detection results, and the improvement methods are summarized. Finally, according to the application progress in nucleic acid, viruses, bacteria, cells, compounds, and heavy metal ions, the development direction of the genetic probe modification of microcantilever sensors is discussed. This review is expected to contribute to the promotion of microcantilever sensor technology.

## 2. Detection Procedure of Genetic-Probe-Modified Microcantilever

The genetic probe is a known nucleic acid sequence with a detection label, which can form a stable double-stranded structure with the sample sequence by complementary binding [13]. The genetic probe can determine the homology degree with a sample sequence by analyzing the optical, electrical, and other generated signals during the complementary binding process. There are four basic conditions for a nucleic acid sequence to be used as a genetic probe [14]:

1. Moderate length. The length of a nucleic acid sequence is generally between 18 and 50 bases. If the sequence is too long, the complementary binding time increases and the detection efficiency decreases; if the sequence is too short, the specificity of detection degenerates;
2. No complementary interval. Nucleic acid sequences with a complementary interval can form a secondary structure by themselves and inhibit the complementary binding;
3. The base composition ratio is constant. The base composition ratio of G and C must be within the range of 40–60%;
4. Avoid multiple iterations of the same base. In order to reduce the false probability, the number of consecutive identical bases should be fewer than four.

**Figure 1:**
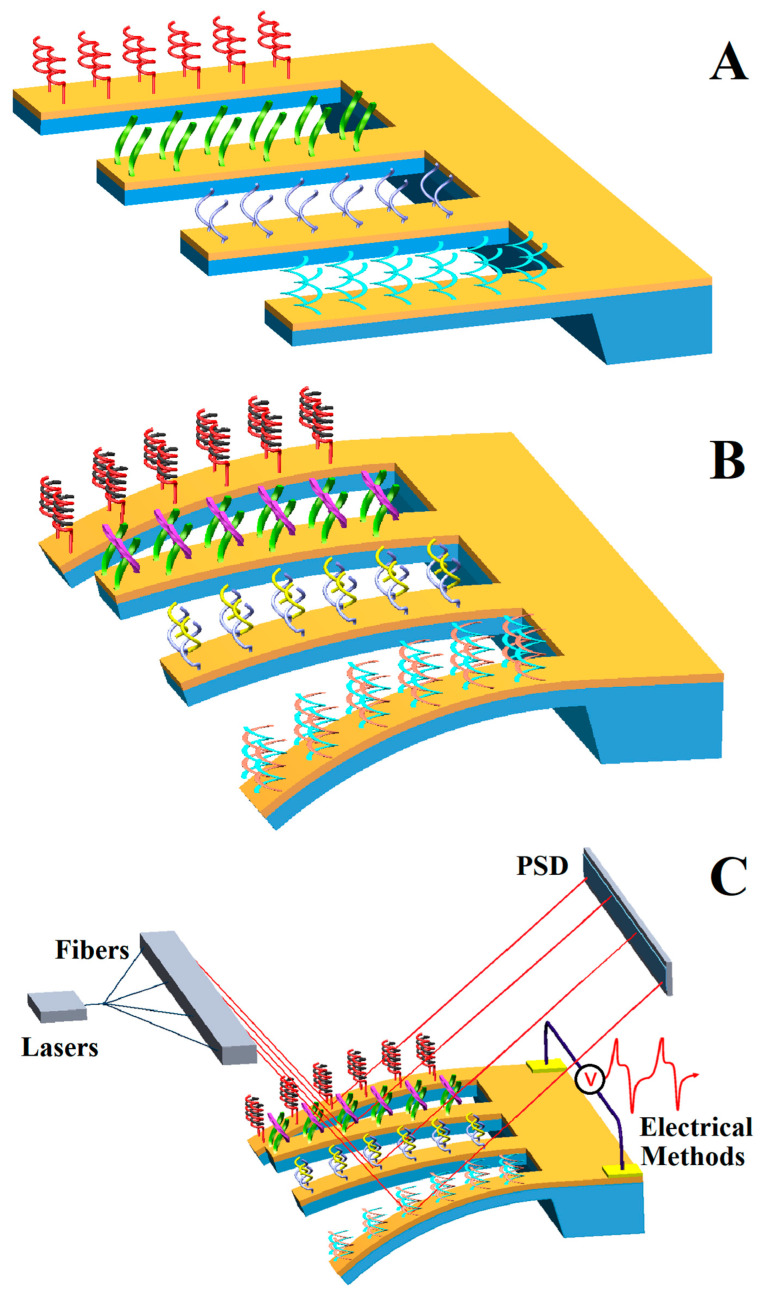
Detection procedure of the genetic-probe-modified microcantilever sensor: (**A**) probe immobilization; (**B**) complementary hybridization; (**C**) signal processing.

Nucleic acid sequences that meet the above conditions can be divided into different types according to the source: DNA [15], oligonucleotide [16], RNA [17], aptamer [18], and so on. These genetic probes are from different sources, and the detection objects and preparation methods are different, but the detection procedure can be summarized as in Figure 1.

### 2.1. Probe Immobilization

In probe immobilization, a nucleic acid sequence with a known order is used as a sensing element to modify the microcantilever surface and form a sensor. Probe immobilization is a key technology of sensor preparation, which determines the specific, proximity, adhesion, and binding force of the sensitive layer to the sample [19]. The immobilization process even has an effect on the stress change of the microcantilever during detection [20]. In order to enhance the stress difference and improve the sensitivity, the genetic probe is usually immobilized on the upper surface of the cantilever free end, while the lower surface is prevented from adsorbing any composition of the sample [21]. The probe immobilization methods include:

**Adsorption.** The genetic probe is combined with the inert carrier on the cantilever surface by interaction caused by hydrogen bonds [22], polar bonds [23], or electrostatic attraction [24]. The adsorption method is convenient and simple, but the reproducibility and storage time are poor because of the reversible adsorption process with environmental sensitivity. Additional chemical treatment [25] or silica nanoparticles [26] can improve the adsorption.

**Entrapment.** The genetic probe is wrapped in polymers [27] or silica gel [28] and then coated on the microcantilever surface [29]. The entrapment method not only immobilizes several genetic probes simultaneously on the microcantilever surface, but also improves the stability of sensitive components. For a polymer-based microcantilever [30], the preparation and modification can be performed simultaneously. However, the carrier material has a certain effect on the mass transfer during the testing process, which can result in an increase in detection time and a decrease in sensitivity.

**Covalent binding.** Covalent bonding needs to be activated by introducing active groups such as carboxyl groups [31], amino groups [32,33], and silane [34] on the cantilever surface or derivative functional groups on the DNA probe [35]. The activation process can improve the firmness and durability of the probe, but it has an effect on the sensitivity and specificity.

**Molecular self-assembly.** The nucleic acid molecules that constitute the genetic probe spontaneously form a highly ordered monolayer on the microcantilever surface and then immobilize [36]. The interaction between monolayer nucleic acid molecules is a noncovalent bond force such as hydrogen bond, Van der Waals force, or π–π stacking effect. At present, molecular self-assembly is one of the most common ways to immobilize the genetic probe, because of the excellent stability and little influence on sensitivity [37]. Before molecular self-assembly immobilization, a gold film is usually precoated on the surface of the microcantilever. The gold film can improve the binding capacity by forming a stable and strong gold–sulfur bond [37,38].

The above immobilization methods can be used in combination to ensure modification efficiency and nucleic acid sequences’ density, as well as prevent modification layer shedding [39]. With the advent of novel micro- or nanofunctionalization processing technologies such as Dip-Pen nanolithography [40], nanoimprint lithography [41], Nano-fountain probe [42], Bioplume TM system [43], inkjet printing [44], microcapillaries [45], and piezoelectric printing [46], genetic probe immobilization technology continues to evolve rapidly.

### 2.2. Complementary Hybridization

Based on the base pairing principles of nucleic acids, A (Adenine)–T (Thymine) and G (guanine)–C (Cytosine), the genetic probe on the cantilever can specifically hybridize to the sample and generate an identification signal. The classical complementary hybridization includes the following types:

**Southern blot** [47]. The Southern blot method was first created and named by E.M. Southern of the University of Edinburgh in 1975. The Southern blot method not only can detect a DNA sample with a homologous fragment and determine the length, but is also effective for recombinant plasmids and phages. The process of Southern blot detection is as follows: extract a DNA sample → electrophoresis → denaturation → transfer membrane → blocking → hybridize to strand → detection. Southern blot is reliable and sensitive but requires radioactive labels such as 32PdNTP and 35SdNTP, and is time-consuming and often fails to give real-time data. Therefore, Southern blot is rarely combined with microcantilever sensors.

**Northern blot** [48]. This method was invented by James Alwine, David Kemp, and George Stark of Stanford University in 1977, and then named “Northern blot” in contrast with Southern blot. The Northern blot method is mainly used for RNA sample analysis. The Northern blot method can detect the exogenous genes that are transcribed in the host and determine the transformed mRNA molecular weight and content. The process of Northern blot detection is as follows: extract a RNA sample → denaturation → electrophoresis → transfer membrane → blocking → hybridize to strand → detection. Similar to Southern blot, the combination of Northern blot and a microcantilever is restricted by radioactive markers.

**Western blot [49]**. The Western blot method, once called immunoblotting, was invented by Harry Towbin of the Michel Friedrich Institute for Biological Research in Switzerland in 1979. The Western blot method, which can recognize proteins from biological tissues’ crude extracts, is mainly for protein molecule detection and analysis. The process of Western blot detection is as follows: extract a protein sample → denaturation → depolymerize (SDS + PAGE) → electrophoresis → transfer membrane → blocking → antibody/antigen reaction → detection. Antigen–antibody reaction is the key step of Western blot and also the main reason that this method is suitable for microcantilever sensors. When the antigen is bound to the antibody, the cantilever bends as a result of the increased intermolecular repulsion between the antigen–antibody complexes [50]. Similar to the antibody–antigen system, the biotin–avidin system can be used to enhance the signal intensity of complementary hybridization. First, biotin is immobilized on the preprocessed surface of a microcantilever by covalent doping or electrostatic adsorption. Then, a nucleic-acid-sensitive layer forms due to the specific affinity between biotin and avidin [51]. The binding force between biotin and avidin is more than six orders of magnitude higher than that of the ordinary antigen–antibody reaction, so the obtained modification layer is more solid and stable [52]. However, the modification procedure is more complicated, and the cost increases correspondingly.

**Figure 2:**
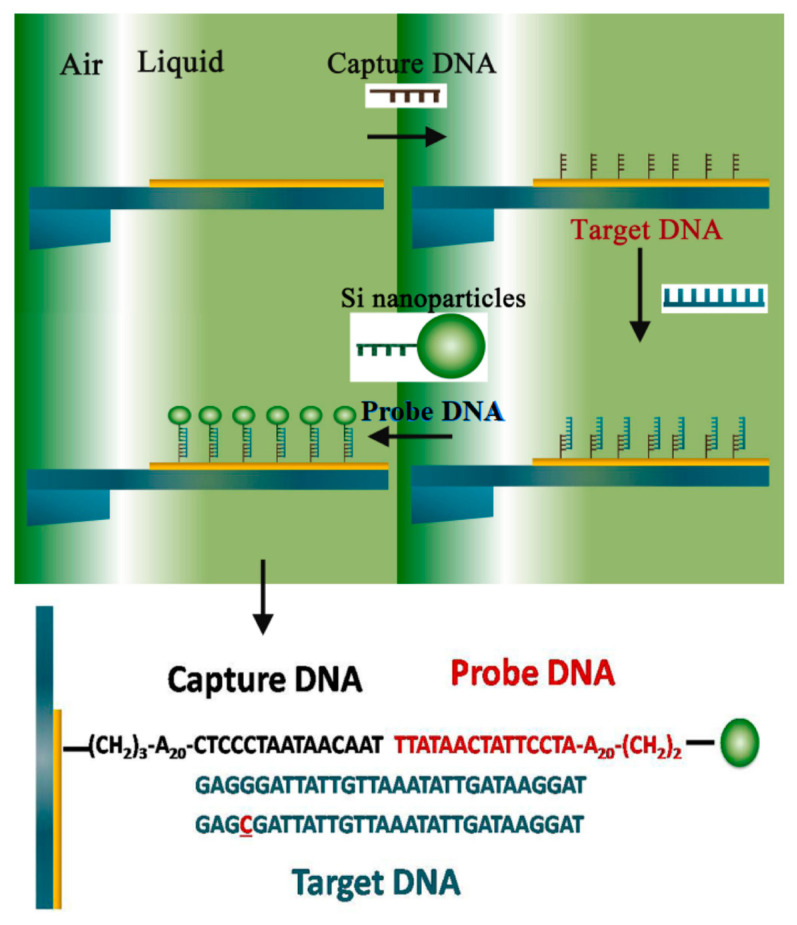
The DNA detection protocol of a spin microcantilever based on in situ hybridization [57].

In addition, according to technical characteristics of detection processes, the complementary hybridization also includes dot blot [53], slot blot [54], and in situ blot [55]. Dot blot and slot blot, named after certain special dosing devices, are rapid quantitative detection techniques derived from Southern blot. In situ blot is a method that combines gene hybridization with histochemistry. In situ blot detection, performed on cell or tissue specimens, can achieve target nucleic acids’ precise localization and quantification [56]. In addition, in situ blot supports nonisotope labeling (biotin, digoxin, and so on), which is characterized by a long storage time and convenient use in detection. The detection of a single-base mismatch using an in situ blot-modified microcantilever is shown in Figure 2 [57]. The spin microcantilever is partly immersed into the liquid, and the captured DNA can be immobilized on the surface. Next, the target DNA and the silica nanoparticle-enhanced probe DNA are added in turn. Meanwhile, the vibrational frequency of the spin microcantilever is measured after each step.

**Figure 3:**
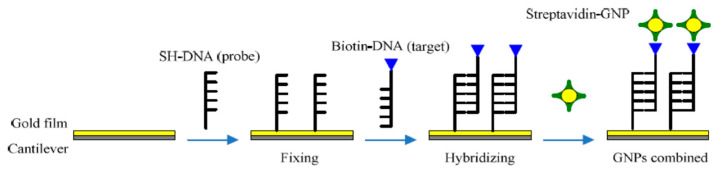
Processes of GNPs combine hybridization information [59].

The specificity of complementary hybridization is excellent and can even achieve single-base mismatch detection. However, the mass sensitivity of the genetic-probe-modified cantilever still needs to be improved due to the nucleic acid being very light weight, especially the short oligonucleotide with almost no weight. As shown in Figure 3, using gold nanoparticles (GNPs) as an additive can improve the mass sensitivity of the cantilever [58,59,60]. First, the cantilever surface is coated with gold film, and then SH-DNA probes are immobilized through the Au–S chemical bond; meanwhile, mercapto hexanol spare parts are shut down on the cantilever surface. After DNA hybridization detection, the sensor passes streptavidin-modified GNPs for an hour. According to the principle of biotin–streptavidin combining, the target’s DNA combines with GNPs while hybridization information amplifies. On this basis, multilevel amplification can also be achieved. The methods that can improve sensor quality sensitivity also include MEMS preparation technology progress, signal extraction method update, and so on.

### 2.3. Signal Extraction and Processing

The molecular recognition signal generated during the complementary hybridization process is converted to microcantilever displacement at the micro- or nanoscale. Appropriate methods of signal extraction and processing are used to extract these small displacements and then convert them to standard signal output by using amplification, filtering, or another method. Strictly speaking, signal processing is not part of a microcantilever sensor; however, the microcantilevers based on the IC fabrication process have a similar preparation process to that of the signal processing circuit, so many microcantilevers have an integration signal processing function [61,62].

**Figure 4:**
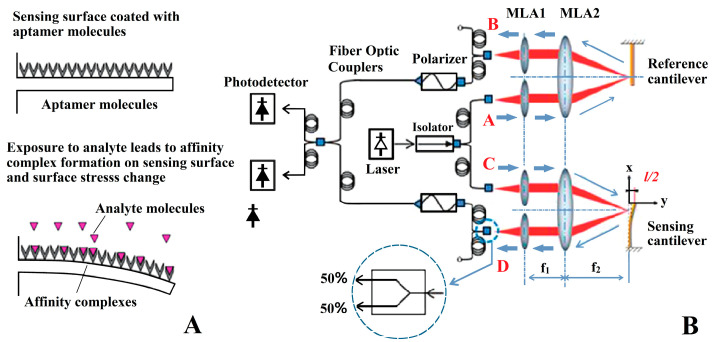
The aptamer-modified microcantilever sensor for cocaine detection. (**A**) Schematic representation of the sensing strategy for cocaine detection. (**B**) Optical circuit of differential surface stress sensor. Laser wavelength is 635 nm. A pair of microlens arrays with lenses of 240 and 900 μm diameter, and pitches of 250 μm and 1 mm, respectively, were used to direct the beams toward the sensing/reference pair [65].

The microcantilever displacement is usually caught by optical or electrical methods. Optical extraction methods can be further divided into optical interference methods [63] that measure the interference fringe displacement between reflected light and reference light, and optical lever methods [64] that amplify microdisplacement by reflecting light. In 2011, Kang et al. [65] proposed an optical differential signal extraction system consisting of two microlens arrays (MLA1 and MLA2) and a sensing/reference microcantilever pair, as shown in Figure 4. The change in surface stress between two microcantilevers can be determined by monitoring the phase difference. Optical differential detection can eliminate the influence of environmental disturbances including nonspecific adsorption, pH, ionic strength, and temperature. When a cocaine-specific aptamer probe is modified on the microcantilever surface, the detection limit of cocaine can be as low as 25 μm (11 mN/m). The optical signal extraction method is derived from AFM. The main advantages of this method are high accuracy and strong anti-interference. However, a detection system based on the optical method requires a CCD camera [66], PSD [67] or tunable laser [68], and precision optical circuit to catch the reflected light caused by the microcantilever, so the system is difficult to miniaturize. However, in 2018, Li et al. [69] fabricated polymer microcantilevers onto the end of standard single-mode fibers using ns-laser machining, which makes the miniaturization of optical detection methods possible, but more reliable immobilization techniques on the cantilever surfaces will be a key issue to solve in the fabrication of these lab-on-fiber biosensors.

**Figure 5:**
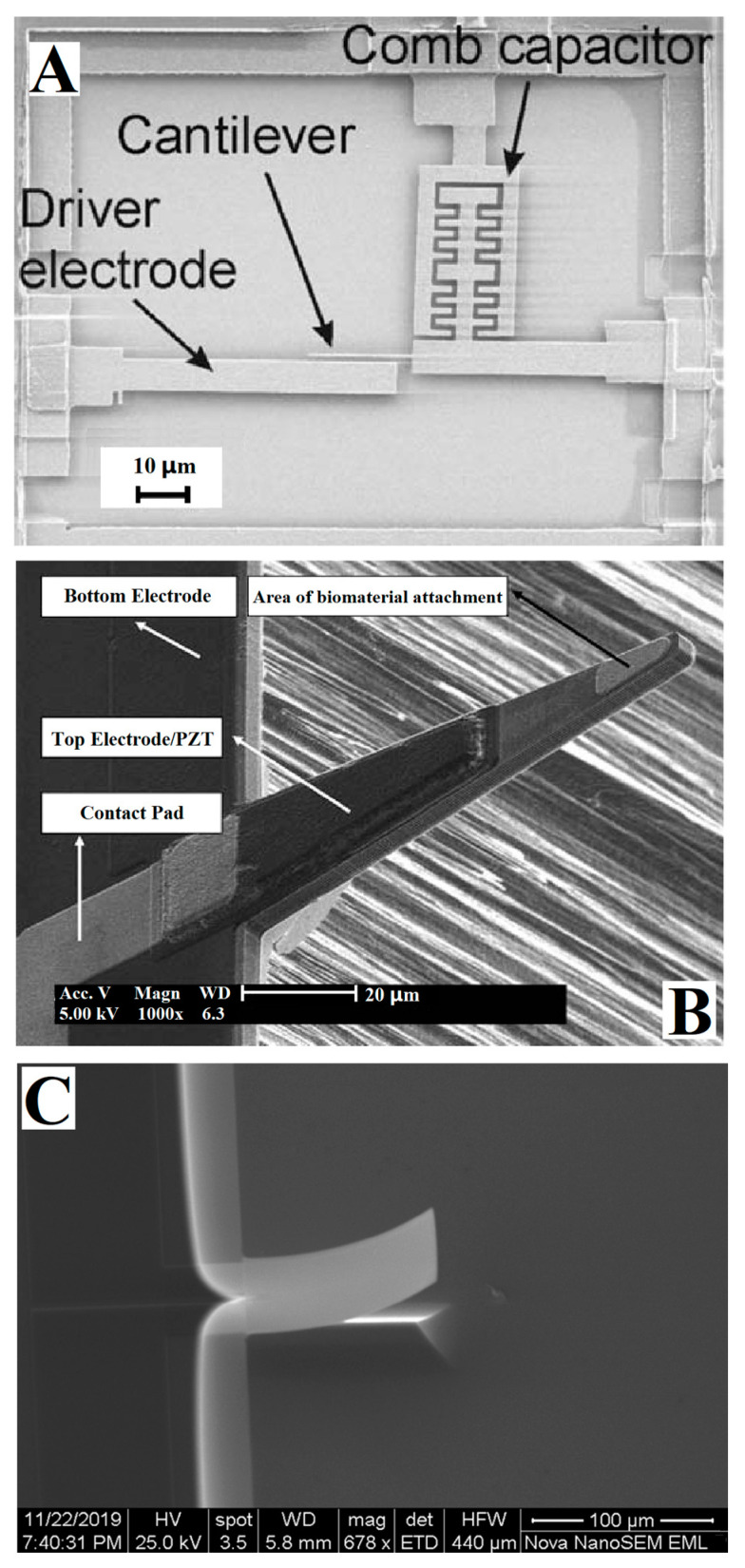
SEM images of microcantilever based on electrical measurement methods. (**A**) Microcantilever with comb capacitance detection, driver electrode, and CMOS signal amplification circuit proposed by Forsen et al. in 2005 [70]. (**B**) In 2007, Lee et al. achieved self-excited dynamic detection of the poly T-sequence DNA and a variety of proteins by using a PZT microcantilever [72]. (**C**) In 2021, Tian et al., developed a polyimide (PI)/Si/SiO_2_ based piezoresistive microcantilever biosensor to achieve a trace level detection for aflatoxin B1 [75].

As shown in Figure 5, electrical measurement methods include: the capacitance method based on a capacitive sensor [70,71] (Figure 5A), the piezoelectric method based on material forward piezoelectric effect [72,73] (Figure 5B), and the piezoresistive method based on material piezoresistive characteristics [74,75] (Figure 5C). The capacitance method has the highest accuracy of the electrical measurement methods. However, the microcantilever with a capacitance sensor is complicated and difficult to assemble. In addition, when used in a liquid environment, the measurement range of the capacitance sensor is narrow and the drift is large. The piezoelectric method is easy to integrate and not limited by the environment. However, the reverse piezoelectric effect is often used to drive a dynamic cantilever. It is difficult to avoid interference when using both the forward and reverse characteristics of a piezoelectric material at the same time. A microcantilever that uses the piezoresistive method is easy to prepare, low in cost, and not as complicated as decoupling methods are, but the detection accuracy is the lowest.

**Figure 6:**
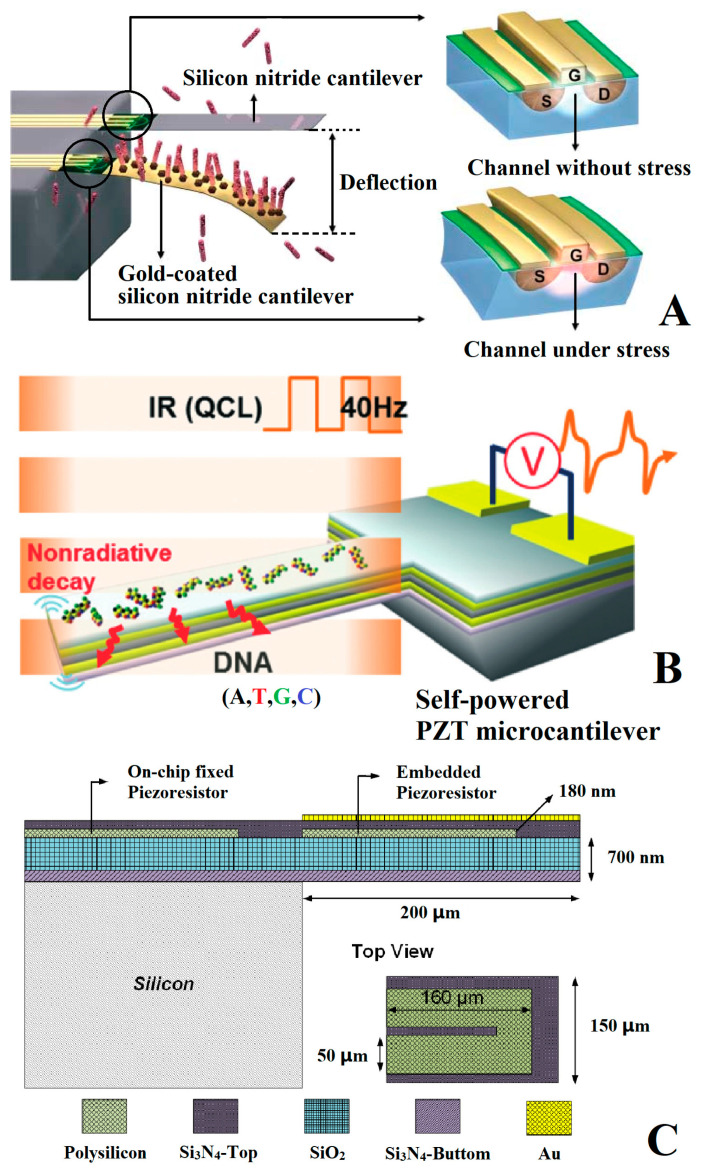
Improved scheme of electric signal extraction method. (**A**) Schematic of the interaction between probe and target molecules on an embedded-MOSFET cantilever system [76]; (**B**) schematic diagram of infrared pyroelectric detection system based on a PZT microcantilever [77]; (**C**) piezoresistive microcantilever sensor chip design with temperature compensation resistance [78].

To enhance electrical measurement methods’ performance, many scholars have proposed improvement schemes. As shown in Figure 6A, in 2006, Shekhawate et al. [76] measured the nanometer-level deflection of a microcantilever by embedding a metal-oxide semiconductor field-effect transistor (MOSFET) into the cantilever to replace the traditional varistor. The silicon nitride cantilever is a reference, and the gold-coated one is used as a sensing cantilever. Due to the low noise, high sensitivity, and direct readout, this approach is suitable for specific binding events with biotin and antibodies detection. Figure 6B shows how, in 2014, Lee et al. [77] reported an optocalorimetric, self-powered sensor for the quantitative detection and discrimination of DNA strands. The piezoelectric and pyroelectric properties of the PZT microcantilever are exploited in the quantitative detection and discrimination of adsorbed DNA strands with their spectral characteristics. The detection limit reached the order of a nanogram. Figure 6C shows how, in 2018, Ku et al. [78] integrated a temperature compensation resistor on the piezoresistive microcantilever, which can reduce the influence of the resistance thermal effect and dual piezoelectric wafer effect from 25.6 μV/°C to 0.3 μV/°C.

**Figure 7:**
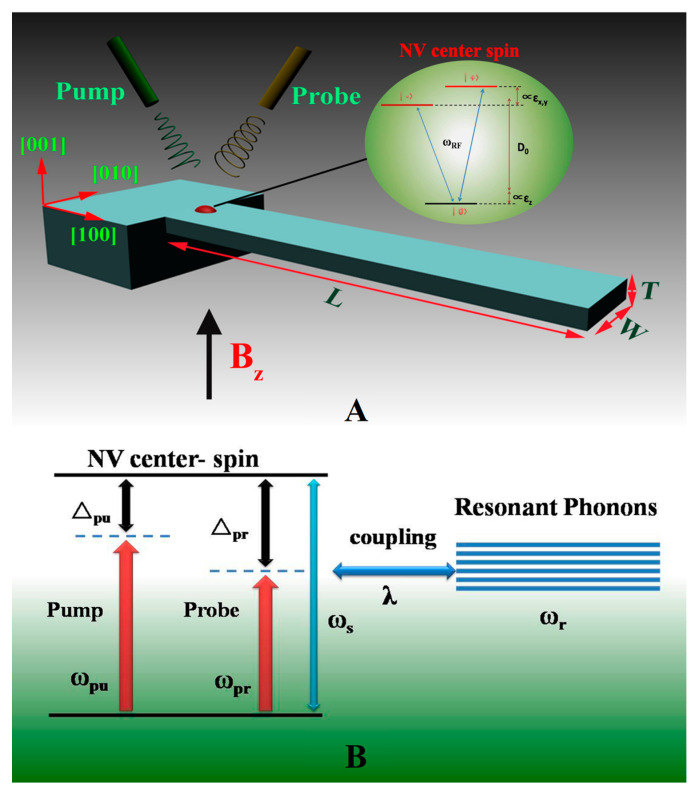
(**A**) Schematic diagram of the hybrid spin microcantilever system in the presence of a strong pump field and a weak probe field; (**B**) the energy level diagram of the coupling between the NV center spin and the microcantilever [57].

In addition to the above optical and electrical methods, the magnetic force also can be used for the ultrasensitive detection of a microcantilever, by establishing a link between very weak molecular interaction and the magnetic force [79,80]. Meanwhile, there are some special signal extraction methods adapted to the genetic probe modification of a microcantilever. As shown in Figure 7, in 2015, Wu et al. [57] combined a nonlinear optical mass sensor using a hybrid spin microcantilever and the nanoparticle-enhanced technique, to detect and monitor DNA mutations. Even one base pair mutation in the target DNA sequence can be identified accurately and in real time.

## 3. Detection Principle of Genetic-Probe-Modified Cantilever

In 1997, Strey et al. [81,82,83] studied DNA double helix interaction force by combining experimental measurement and theoretical analysis, which was the foundation for the signal conversion mechanism of a genetic-probe-modified microcantilever. In order to eliminate interference from the external environment during the detection, it is necessary to find ways to further improve sensitivity, optimize the genetic-probe-modified microcantilever design, and accelerate its practical applications, and much theoretical research must be done, including study relating to the deflection or frequency shift mechanism with probe immobilization or hybridization action on the microcantilever surface as well as analyzing the effects of DNA molecular density, chain length, and conformational entropy on the mechanical response of the microcantilever.

### 3.1. Deflection Principle

In 2000, Fritz et al. [21], the first group that immobilized a genetic probe on a microcantilever, attributed the deflection to differential stress between the modified and unmodified surfaces of the microcantilever. In 2001, Wu et al. [84] studied the origin of surface-stress change by using DNA hybridization experiments on a cantilever. The cantilever deflection lies in the interplay between changes in configurational entropy and intermolecular energetics induced by specific biomolecular interactions (such as DNA–RNA, antigen–antibody, protein–ligand, and DNA–protein), and the deflection direction can be changed by adjusting the configurational entropy and intermolecular interactions. In 2002, in subsequent research [85], they proved that the direction or magnitude of microcantilever deflection can be manipulated by adjusting the configuration entropy or intermolecular interactions (such as the number of mismatch points). Also in 2002, McKendry et al. [86] suggested that the nanomechanical motion of a microcantilever originates predominantly from steric hindrance effects and depends on the concentration of DNA molecules in solution. In 2003, Liu et al. [87] proposed that the nanomechanical bending of a cantilever is caused by the flexoelectric effect [88], instead of the conformational entropy force suggested by Wu et al. Based on the above theory, they verified an apparent semimicroscopic relationship between cantilever deflection, ssDNA length, and salt concentration, but could not define the percentage of ssDNA probe hybridization. In 2004, Alvarez et al. [36] studied the interaction forces responsible for microcantilever bending when the self-assembled monolayer hybridizes with the complementary nucleic acid. They concluded that the main source of surface stress during the immobilization is the covalent bond between the surface gold atoms and the sulfur atoms of the thiol linker of the DNA probes, while the only contribution to the surface stress during hybridization is intermolecular forces between neighboring DNA molecules. In 2006, Huber et al. [89] believed that the surface stress generated when transcription factors bind to specific sites on double-stranded DNA oligonucleotides is the cause of cantilever deflection. In the same year, Stachowiak et al. [90] found that the cantilever surface stress generated by the complementary hybridization process is a combined effect of DNA strand length, graft density, and hybridization efficiency. In 2009, Arroyohernández et al. [20] observed the immobilization process of the ssDNA-modified layer using X-ray diffraction and X-ray photon spectroscopy, and they found that the sign of microcantilever stress difference is determined by the changes in structure and kinetic energy during S–Au bonding. In 2010, Godin et al. [91] explored various mechanisms associated with molecular adsorption on the cantilever surface and their impact on induced surface stresses. The results showed that the surface stress resulting from adsorption-induced changes in the electronic density of the underlying surface is up to 2–4 orders of magnitude larger than that resulting from intermolecular electrostatic or Lennard–Jones interactions.

### 3.2. Detection Model

Based on the above deflection principle, many scholars have proposed semiquantitative or quantitative models to explain and predict the response changes of a cantilever sensor. In 2002, Hagan et al. [92] presented a model that can account for the cantilever deflections resulting from the adsorption and subsequent hybridization of DNA molecules. In the model, the forces determining equilibrium cantilever deflection can be divided into electrostatic free energy (FELEC), free energy resulting from macromolecular conformational entropy and nonelectrostatic interactions (FPOLY), free energy contribution associated with the osmotic pressure of the counterions (FOSM), and a mechanical energy penalty associated with bending the cantilever (ECANT). For dsDNA, the model results showed that the dominant factor determining cantilever deflections is hydration force, not electrostatics or conformational entropy; for ssDNA, the adsorption deflection is smaller than that for dsDNA, which agrees with Fritz et al. [21] but is not consistent with Wu et al. [84]. They also used the model to highlight the importance of grafting densities and found the influence of disordered grafting points on deflection. However, a model calculation based on molecular equilibrium adsorption is not relevant to nonspecific segment/surface interactions.

In 2006, the group of Zhang [93,94] started work on a model of stresses in a multilayer microcantilever. In 2007, they proposed a laminated cantilever model combining a piezoelectric biolayer in continuum mechanics, the linearized Poisson–Boltzmann equation from statistical mechanics, and the scaling method from polyelectrolyte brush theory [95]. In this model, the cantilever consists of four layers, including a Si layer, a Ti layer, an Au layer, and a ssDNA molecular brush biolayer immobilized by self-assembly of the thiol group. They analyzed the relationship between the nanomechanical deflection of the cantilever and factors such as the nanoscope structural features of ssDNA molecules, buffer salt concentration, and macroscopic mechanical/piezoelectric parameters of DNA probes using this model, and concluded that the piezoelectric effect of the biopolymer brush layer is the main factor in cantilever nanomechanical bending. In 2008, the group introduced the DNA liquid crystal equation [83] into the above four-layer microcantilever model, and proposed an energy model for the nanomechanical study of cantilever–DNA deflection [96]. This model also considers the contribution of the normal strain at the centroidal principal axis to the chip deflection. The numerical results showed that cantilever deflection enhances with an increase in length of DNA chains, and the interchain distances should be carefully controlled to no less than 4 nm during the process of probe molecule self-assembly. In 2009 [97], by revising the analytical Stoney’s equation, they presented an alternative two-variable model to formulate the overall free energy of dsDNA probes. The model included electrostatic energy between neighboring strands, hydration energy between DNA molecules and hydrogen bonding networks in water, and conformational fluctuations of dsDNA. The computation results showed that predictions by the first-order approximation are in good agreement with the experimental data from Stachowiak [90] in a 1.0 M sodium phosphate buffer. In 2009 [98], the group investigated the influence of the hybridization exothermic effect on the nanomechanical deflections of a cantilever by using an alternative model for the thermoelasticity of a laminated cantilever. In 2010 [99], they studied the influence of screened electrostatic repulsion in the microcantilever DNA grafting process by combining their alternative two-variable model [97] with the sphere–chain model for ssDNA [92]. The model computation results from the Monte Carlo method showed that the cantilever deflections grow with the increase in grafting density or nucleotide number. In 2011 [100], in order to reveal the relationship between nanomechanical packing deflections, based on the model in [81,87], the group formulated a four-layer model for cantilever deflection by inducing a counterion osmotic effect. The model showed that the contribution of the normal strain at the centroidal principal axis to the cantilever deformation and the contribution of the Au and Cr layers to the mechanical energy of the cantilever cannot be neglected. Moreover, the packing deflections grow with an increase in grafting density, chain length, or buffer salt concentration. In 2013 [101], to describe the nonuniformity of DNA probe thickness, the group improved the empirical model for osmotic experimental systems [81,82,83] under the net charge assumption. The parameters of the new multiscale model are from the curve fitting of the experimental data, which can predict the inhomogeneity and elastic properties of a DNA biofilm on a microcantilever. In 2015 [102], to refine the above multiscale model [101], the group carried out a microcantilever bending experiment and obtained the empirical parameters of interactions in ssDNA film. By comparison with the simulation results, it was found that the average spacing assumption of the traditional DNA stacking model greatly underestimated the film elastic modulus. In 2021, the latest research results of Zhang [103] led to an alternative mechanical model to characterize the clamped-end effect on the static detection signals of the DNA microcantilever. In this model, the clamped-end effect on the static deflection signals is discussed, and the importance of the neutral axis shift effect is also illustrated for an asymmetric laminated microcantilever.

In addition to the above studies, in 2007 Merlo et al. [104] investigated the electrostatic field within DNA molecules and its force consequently acting on the cantilever by establishing a cylinder model based on the electrostatic potential of dsDNA molecules arrays immersed in an ionic solution. In 2008, Sushko et al. [105] reported the first quantitative multiscale model to describe the transduction of specific biochemical reactions into micromechanical cantilever bending motion. Considering the effects of the chemical, elastic, and entropic properties of the cantilever material and sensing layer, the model can predict the cantilever deflection direction and magnitude upon pH variations of the buffer solution and for various chain lengths of SAMs. Another achievement of this model is identifying the biaxial elastic modulus of the sensing layer for improved detection sensitivities. In 2009, to study the chemomechanical response origin of cantilever arrays, Sushko et al. [106] proposed a quantitative mesoscopic model that includes two competing components to the differential deflection: a specific chemical or physical reaction on the active cantilever and the elastic property difference of the active or reference coatings. In 2010, to predict dsDNA surface coverage and induced surface stress, Huang et al. [107] proposed adsorption and interaction models that can provide a clear quantitative explanation for the practical limit on the number of base pairs.

### 3.3. Impact of Environmental Factors

From the research results of the deflection principle and detection model, it can be seen that the microcantilever deflection is affected not only by sample concentration, sensor hierarchy, DNA probe grafting density, chain length, and other factors, but also by many environmental factors.

#### 3.3.1. Temperature

In 2002, Yue et al. [108] used chip-scale high-throughput microcantilever arrays to study the effect of detection time on thermomechanical sensitivity. The results showed that the long-term drift of microcantilevers can reach 2.1 nm/min, so the test should be completed within 10 min. In 2006, Biswal et al. [109,110] found that, after heating, the dissociation and melting processes of the dsDNA-modified layer would lead to changes in the electrostatic, anti-ionic, and hydration forces between the remaining DNA molecules on the microcantilever surface. Furthermore, the melting temperature of the dsDNA modification layer is a function of chain length and salinity. In 2013, Joseph et al. [111] studied the thermal induction influence on local and global structures by modifying the DNA molecule with a hairpin structure on the surface of the microcantilever. The results showed that the microcantilever deflection curve is a function of temperature.

#### 3.3.2. Salinity

In 2003, by inducing a microcantilever deflection experiment based on DNA adsorbing hybridization, Liu et al. [87] verified that there is a semimicroscopic relationship between the amount of microcantilever deflection, the length of ssDNA modification layer, and the concentration of buffer salt. In 2006, Stachowiak et al. [90] found that, by changing the ionic strength of a solution, the grafting density of the DNA-modified layer can be controlled, thus establishing a relationship with the surface stress. In the same year, Zhang et al. [93,95] studied the effect of the concentration and type of buffered salt solution on the microcantilever’s deflection. The results showed that the osmotic pressure generated under high salinity contributed much to the deflection of the microcantilever, but the deflection direction would be decided by the competition between salt ions, H^+^, and OH^−^. In 2011, Mertens et al. [112] found that the NaCl concentration has a certain influence on the interaction between ssDNA-modified microcantilevers and spermidine. With a decrease in NaCl concentration, the surface stress changes from repulsion to gravitation, and the value increases gradually. In 2017, Wu et al. [113] pointed out that, due to the competition between microgravity and repulsion, the piezoelectric coefficient of the low-density dsDNA film in the multivalent salt solution is negative, while the piezoelectric coefficient of the high-density film is positive. In addition, the piezoelectric coefficient change is closely related to the microcantilever signal.

#### 3.3.3. Humidity

In 2008, Mertens et al. [114] studied the influence of humidity on a ssDNA-modified microcantilever by controlling the proportion of dry and wet nitrogen in the environment. The results showed that water adsorption plays an important role in the process of ssDNA membrane self-assembly. In 2014, Domínguez et al. [115] investigated the influence of humidity on the swelling and ablation process of the self-assembled DNA modified layer. It was found that the compressive stress on the microcantilever surface is proportional to the humidity, and reaches a peak when the humidity is 2–3%. In 2017, this research group [116] also found that the Young’s modulus of the ssDNA film is proportional to humidity and reaches a peak value when the grafting density is 3.5 × 10^13^/cm^2^.

#### 3.3.4. pH

In 2005, Shu et al. [117] found that the motion direction and amplitude of a microcantilever with an integrated DNA motor can be adjusted by the pH value and ionic strength of the buffer solution, and there is a sharp change in the surface stress direction of the microcantilever when pH = 6.7. In 2012, Zhang et al. [118] investigated the pH effect on the hybridization efficiency by using high-density ssDNA modification of a microcantilever. The results showed that the hybridization efficiency is lower at a lower pH (4.5) and reaches a peak at a higher pH (7.5–8.5). In 2017, the same group [119,120] studied the effects of pH on the electrical and mechanical properties of DNA films. They found that the competitive relationship between H^+^ and OH^−^ determines the microcantilever deflection direction, and the uniformity of ion distribution also affects the stiffness of DNA film (the film is softer under acidic conditions).

## 4. Application of Genetic-Probe-Modified Cantilever

In 2000, a group led by Fritz [21] published an article in *Science* about using genetic-probe-modified microcantilevers for the first time to detect oligonucleotides. In the following 20 years, genetic-probe-modified microcantilevers have achieved fruitful applications in the fields such as DNA, RNA, viruses, bacteria, proteins, and trace ions.

### 4.1. Nucleic Acid

**Figure 8:**
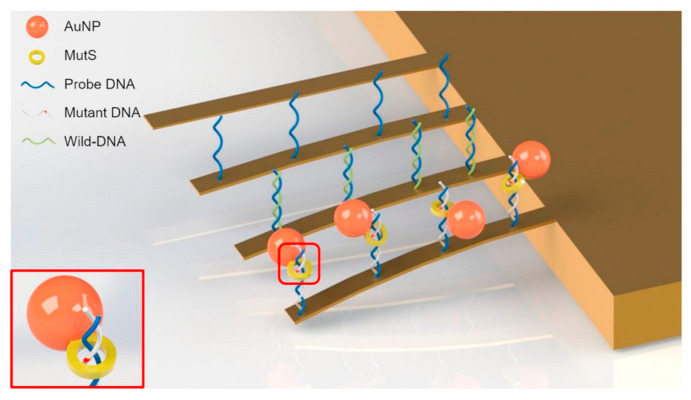
Schematic illustration of the Kirsten rat sarcoma viral oncogene homolog (KRAS) mutation detection using MutS, AuNP, and resonator [127].

In 2002, the McKendry group of IBM [86] reported a microcantilever array that can sequence-specifically detect unlabeled DNA targets in 80-fold excess of a nonmatching DNA background solution. This proves the excellent anti-interference ability of a genetic-probe-modified microcantilever. In 2003, Su et al. [121] detected DNA strands by using a microcantilever with gold-nanoparticle-modified genetic probes in dynamic mode. After the amplification process, by catalyzing the nucleation of silver, the method can detect target DNA at a concentration of 0.05 nM or lower. In 2005, Ilic et al. [122] further improved the dynamic detection sensitivity by using scanning optical–thermomechanical motion excitation method. The sensitivity of their cantilever array was sufficient to detect the binding of a single large biomolecule without labeling. In 2006, Zhang et al. [123] first demonstrated the nanomechanical analysis of multiple differential gene expression of 1–8U, a potential marker of cancer progression or viral infection, by using cantilever–array sensors in a complex background without amplification or labeling. Also in 2006, Huber et al. [89] investigated the interaction between dsDNA and two different DNA-binding proteins, the transcription factors SP1 and NF-κB, by using cantilever arrays. This demonstrated the feasibility of micromechanical cantilever sensors for investigating transcription factors. In 2007, Kishan et al. [124] successful detected small DNA sequences at a femtomolar concentration in human serum by using a 15-mer ssDNA-modified piezoelectrically excited cantilever. In 2010, the group of Miyachi [125] reported a method of systematic evolution of ligands by an exponential enrichment (SELEX) using a cantilever based on AFM to obtain aptamers that have a strong affinity for target molecules. Thrombin, at concentrations as low as 0.2 nM, can be detected by the AFM-SELEX method [126]. In 2014, Mishra et al. [44] modified short nucleic acid sequences onto the microcantilever array surface by using inkjet printing technology. This method improved the detection sensitivity of single-chain peptide nucleic acids (PNA) by about 20-fold, and the detection limit reached the single-base misalignment level. As shown in Figure 8, in 2019, Park et al. [127] modified microcantilever arrays using DNA probes, Au nanoparticles, and mismatch recognition proteins (MutS). Single-nucleotide polymorphisms (SNPs) of cancer markers can be successfully realized with a detection limit of 100 fM using this method. In addition, to trace detection of nucleic acids and antibodies, a microcantilever modified by genetic probes can also be used for research into DNA enzyme digestion [128] or DNA strand elasticity [129].

### 4.2. Viruses, Bacteria, and Cells

#### 4.2.1. Viruses

A virus is an acellular organism that contains only nucleic acid (DNA or RNA), parasitizes cells, and proliferates by replication. Viruses are tiny but harmful, and they have caused global public health problems many times. Therefore, viruses are among the main detection objects of a genetic probe modification microcantilever.

**Figure 9:**
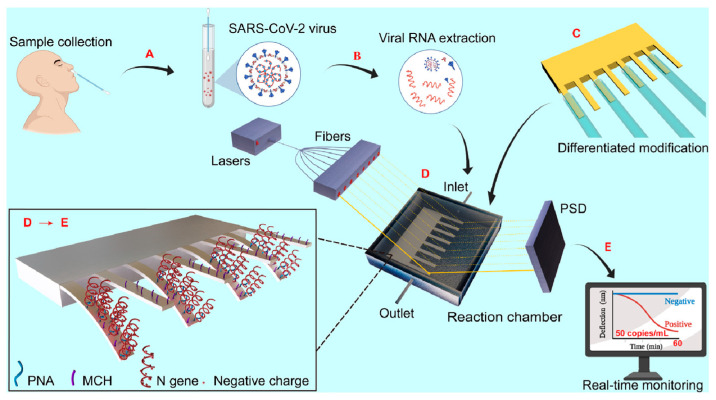
Schematic of the nanomechanical method of SARS-CoV-2 detection from sampling to diagnosis. (**A**) Sample collection from infected individuals; (**B**) RNA extraction of SARS-CoV-2; (**C**) differential modification of microcantilever array with PNA. Four microcantilevers in an array were functionalized with PNA, and the other four were used for in situ comparison. (**D**) Detecting with nanomechanical devices. Eight semiconductor lasers sequentially emitted a stable beam focused on the tip of each microcantilever in the array, while a position-sensitive detector (PSD) was responsible for monitoring the deflection of each microcantilever in real time by measuring the movement of the reflected light. (**E**) Early diagnosis of COVID-19 within 60 min [137].

In 2006, Sreepriya et al. [130] detected feline coronavirus at a concentration of 0.1 μg/mL using a microcantilever modified by feline coronavirus antiserum. The results confirmed the suitability of a genetic-probe-modified microcantilever for the detection of severe acute respiratory syndrome-associated coronavirus (SARS-CoV). In 2007, Hwang et al. [131] used an RNA aptamer as a microcantilever sensitive layer, and successfully detected hepatitis C virus (HCV) at a concentration of 100 pg/mL. In 2009, Kim et al. [132] introduced an in/ex situ monitoring method of the HBV by using PZT-embedded microcantilever sensors. The DNA probe (37-mer including T10 spacers) specific to HBV DNA is immobilized on the gold-coated microcantilever to achieve the recognition layer. Moreover, to increase the DNA binding efficiency, an ethylene glycol spacer (HSC11-EG3-OH) is backfilled on the modification layer. In 2010, Cha et al. [133] reported a dynamic microcantilever biosensor for HBV DNA detection. When using silica nanoparticles (SiNPs) containing rhodamine B isothiocyanate (RITC) for signal amplification, the detection limit of target HBV DNA (243-mer nucleotide) was found to be up to the femtomolar level. In 2012, Shu et al. [134] successfully detected the grouper nerve necrosis virus using a silicon nitride microcantilever modified by antimicrobial peptides (AMPs). In 2013, Abdullah et al. [135] successfully detected target ssDNA and ssRNA in human immunodeficiency virus (HIV) by using a silicon-based microcantilever modified by mercapto-oligonucleotides. In 2015, Kim et al. [136] used the specific primer of human papilloma virus (HPV) as the sensitive layer of silicon-based varistor microcantilever and combined it with PCR amplification to successfully detect HPV. In 2022, Wang et al. [137] developed an ultrasensitive nanomechanical method based on a microcantilever array and PNA probes for the detection of SARS-CoV-2 virus. The detection process is described in detail in Figure 9, while the method has an extremely low detection limit of 0.1 fM (105 copies/mL) for an N-gene-specific sequence (20 bp). In addition to virus detection, with the help of an AFM system, a microcantilever can also carry out a series of operations such as imaging, operation, and transmission monitoring of a single virus [138].

#### 4.2.2. Bacteria

The structure of bacteria is much more complicated than that of viruses. For molecular biology testing, specific nucleic acid fragment sequences of pathogenic bacteria are the detection target of a microcantilever.

In 2013, Rijal et al. [139] detected *Escherichia coli* O157:H7 (EC) in beef samples by using a piezoelectrically excited cantilever with the toxic gene *stx2* as a sensing layer. Compared to the traditional antibody–antigen method (2500 cells/mL), a much lower concentration can be detected by this method without any culture enrichment or amplification (under 700 cells/mL). In 2014, Xu et al. [140] used porous silica functionalized with NH2 as the medium layer of a piezoresistive microcantilever, then the streptomycin avidin was blocked with bovine serum albumin to form a sensitive layer on the medium layer. The rapid real-time detection of *Escherichia coli* was successfully realized by using the enzyme cleavage reaction between the sensitive layer and gene stx2 of O157:H7. In 2019, Zheng et al. [141] fabricated a gold-nanoparticle-amplified microcantilever array biosensor that can determine in parallel ultralow concentrations of foodborne bacteria, including *Escherichia coli* O157:H7, *Vibrio parahaemolyticus*, *Salmonella*, *Staphylococcus aureus*, *Listeria monocytogenes*, and *Shigella*.

**Figure 10:**
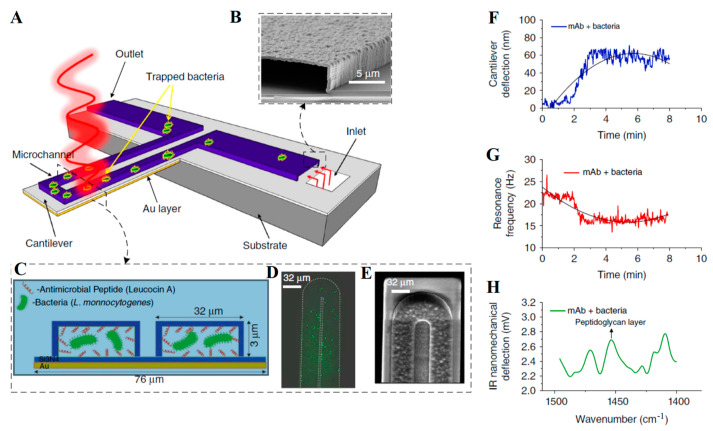
A schematic representation of the BMC and its multimode operation [143]. (**A**) BMC filled with bacteria supported on a silicon substrate; (**B**) SEM image of the cross section of an inlet; (**C**) cross section of microchannel on BMC modified with mAb or AMP; (**D**) fluorescent image from the top side of the BMC, filled with bacteria; (**E**) SEM image of the tip of the BMC; (**F**) deflection of BMC caused by heat when bacteria absorb infrared light; (**G**) resonance frequency changes with the quality of bacteria; (**H**) selective absorption of infrared light by bacteria.

In addition to *Escherichia coli*, in 2015, Khemthongcharoen et al. [142] detected *Vibrio cholerae* by combining a gold-coated piezoresistive microcantilever with a self-assembled monolayer (3-mercaptopropionic acid (MPA), for the immobilization of a specific DIVA probe via avidin). As shown in Figure 10, in 2016, Etayash et al. [143] modified a bimaterial dynamic microcantilever (BMC) with monocyte proliferation monoclonal antibody (mAb) or monocyte proliferation, targeting antibacterial peptide (AMP). With the help of a microfluidic channel integrated on the microcantilever, not only was the detection of *Listeria monocytogenes* successfully realized, but the response of bacteria to antibiotics could also be monitored in real time. In 2021, Wang et al. [144] combined an antibody-modified microcantilever with AC thermoelectric technology, which improved the capture efficiency of *Vibrio parahaemolyticus* and shortened the detection time. Recently, *Yersinia* [145] and *Mycobacterium tuberculosis* [146] were also successfully detected by a genetic-probe-modified microcantilever sensor. In addition to detection and drug interaction research, a microcantilever can also be used for bacterial growth monitoring [147].

#### 4.2.3. Cells

Cells are the basic structural and functional units of an organism. All organisms except viruses are known to be made up of cells. The structure of cells is complicated, so the targets are specific nucleic acid sequences or another biomarker when detecting cells by a microcantilever.

Cancer cells are one of the highest detection priorities of microcantilever sensors. In 2006, Dell’Atti et al. [24] immobilized the biotin probe on the surface of a piezoelectric microcantilever through a “glucan–streptomycin” avidin medium layer, and successfully detected mutations in the *TP53* gene in leukemia cells. In 2010, Ricciardi et al. [148] used receptor–ligand and antibody–antigen systems to modify a dynamic microcantilever, and successfully detected the angiogenesis marker Ang-1 of cancer cells. The results also showed that the antibody–antigen method is more advantageous. In 2011, Loo et al. [73] detected HER2, a biomarker commonly overexpressed in the blood of breast cancer patients, using a magnesium niobate–lead titanate/tin piezoelectric material microcantilever (PEMS). This was the first report of the detection of naturally occurring cancer biomarkers in serum by a cantilever. In 2014, Le et al. [149] used a silicon nitride microcantilever with a self-assembled monolayer to detect Golgi protein 73, which is a serum biomarker used for diagnosing human hepatocellular carcinoma. The concentration detected was up to 400 ng/mL.

**Figure 11:**
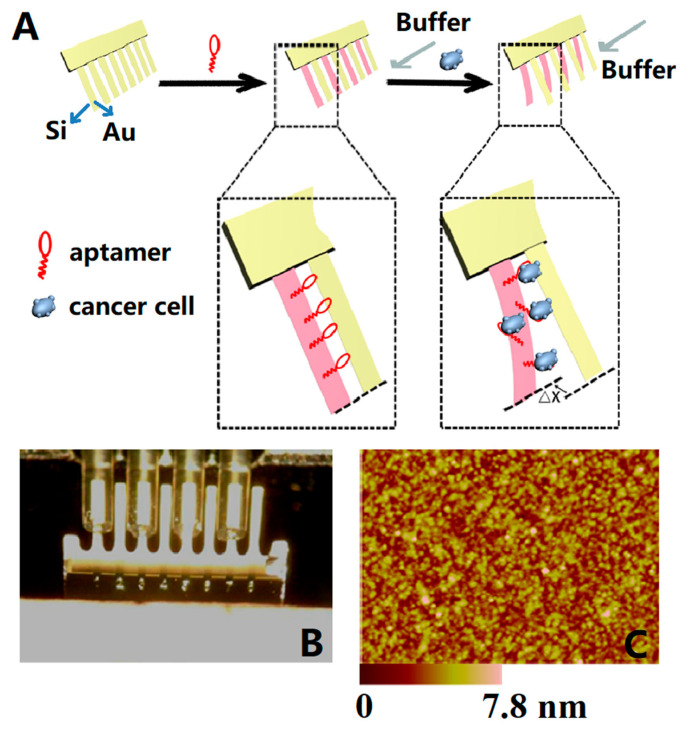
(**A**) A schematic illustration of the mechanism of *HepG2* cells determined by a microcantilever array sensor; (**B**) functionalization procedure of microcantilevers by immersing into capillaries containing TLSIIa aptamers; (**C**) AFM topography image of aptamers (1 μmol/L) on gold surface (2 × 2 μm) [45].

In recent years, with the development of MEMS technology, microcantilevers have been able to detect intact cells. In 2015, Etayash et al. [150] demonstrated a microcantilever that functionalized with a cancer-specific peptide 18-4 (WxEAAYQrFL) and showed significant deflection on breast cancer cell (MCF7 and MDA-MB-231) binding. The detection limit was 50–100 cells/mL, and the capture yield was 80%. In 2016, Chen et al. [45] developed a microcantilever array with a TLSIIa aptamer probe for label-free detection of liver cancer cells (HepG2). As shown in Figure 11A, four microcantilevers were modified with aptamers as sensing microcantilevers (pink), and the other four as reference ones (yellow). Δx indicates the differential signal induced by the interaction between aptamers and HepG2 cells. The gray arrow indicates the flow direction of the binding buffer. The detection linear relation ranged from 1 × 10^3^ to 1 × 10^5^ cells/mL, with a detection limit of 300 cells/mL (S/N = 3).

**Figure 12:**
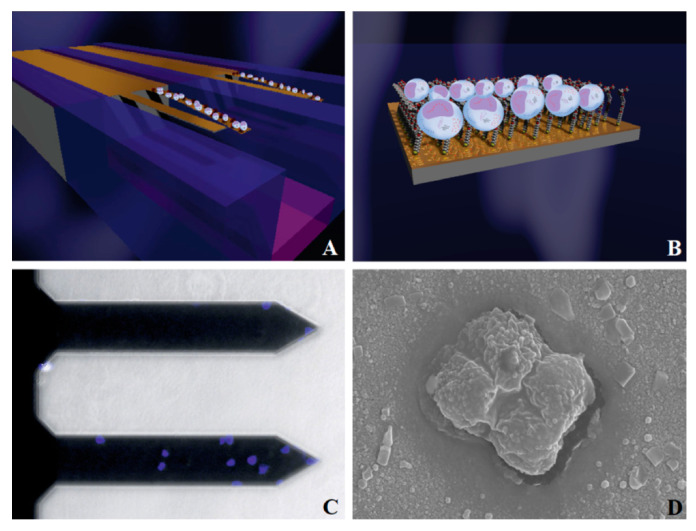
Nanomechanical detection of cancer cells in a model of breast cancer. (**A**) Schematic diagram showing the attachment of malignant cells to the cantilever surface; (**B**) close-up image of the cell–receptor complex on the nanomechanical cantilever surface; (**C**) attachment of stained MDA-MB231 breast cancer cells (blue) on the working microbeam and the reference microbeam; (**D**) SEM of a cancer cell attached to the measuring nanomechanical cantilever sensor [151].

As shown in Figure 12, in 2018, Etayash et al. [151], successfully detected breast cancer cells (MDA-MB231) by using a microcantilever array composed of a decapeptide-modified working sensor and a 6-hydroxy-1-hexanethio-modified reference sensor. The research group also used normal mammary epithelial cells (MCF10) as a control group, changing the arrangement of modified probes to study the differences in signal pathways between cancer cells and normal cells. In addition, the microcantilever can be used to study the dynamic deformation difference between cancer cells and normal cells, and the results may indicate a new potential marker to identify cancer cells [152].

### 4.3. Other Substances

#### 4.3.1. Proteins

Proteins, which are composed of various amino acid molecules in proportion, are not only an important component of human cells and tissues, but also an important participant in life activities through constant metabolism and renewal in the body.

**Figure 13:**
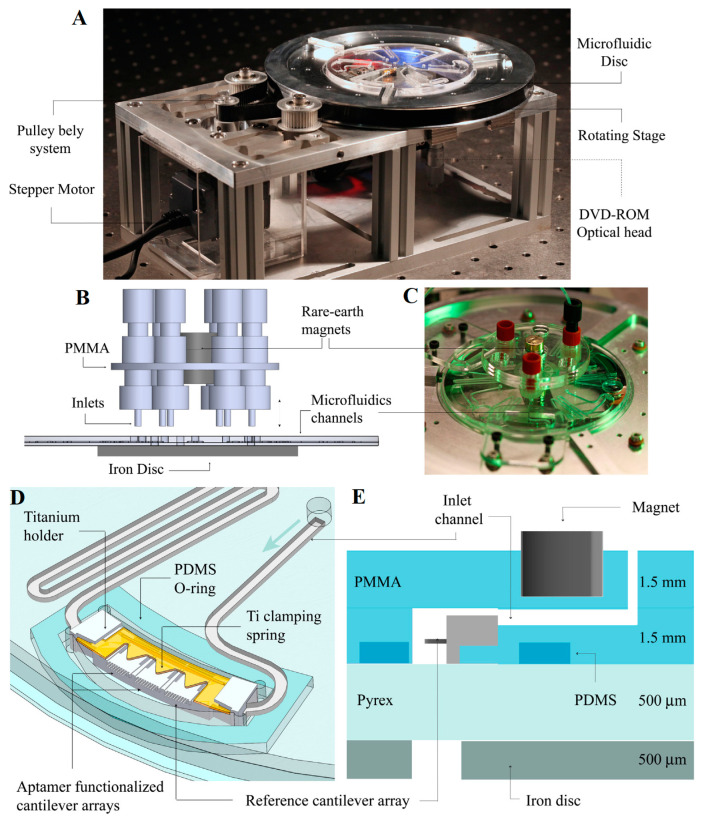
(**A**) Picture of the complete setup; (**B**) schematic view of magnet-based microfluidic inlet assembly; (**C**) a polymer disc connected to nozzles through rare earth magnets; (**D**) installation schematic of the chip on the titanium alloy bracket; (**E**) installation schematic of polymer chip composed of PDMS and PMMA [156].

In 2004, Savran et al. [153] modified the aptamer probe on the working cantilever surface while there was a nonspecific oligonucleotide probe on the reference cantilever, and successfully recognized specific proteins containing thrombin and Taq DNA polymerase. In 2007, Yoo et al. [154] modified an avidin-sensitive membrane on the surface of a microcantilever by self-assembly and monitored in real time the binding process of a streptavidin ligand. In 2010, Wang et al. [155] used a microcantilever modified by a platelet-derived growth factor (PDGF) aptamer probe to quantitatively study the effect of temperature on the binding process between the probe and PDGF. As shown in Figure 13, in their subsequent research from 2013, they integrated a microcantilever array modified by a PDGF aptamer on the microfluidic chip, and built a plug-and-play detection platform with a DVD-ROM as the optical detection module, which can realize fast, low-cost, and parallel detection [156]. In addition, in 2012, Zhai et al. [157] modified a microcantilever with RNA aptamers to detect lipid carrier protein (Lipocalin-2). The system showed a detection limit of 4 nM, and the study results also demonstrated that the RNA aptamer can bind to the siderophore binding pocket of the protein. In 2015, Liu et al. [158] modified a dynamic microcantilever with the biotin–antibiotin system to detect ricin protein. In 2018, the group of Agarwal [159] detected a heart-type fatty acid-binding protein (h-FABP) in a trace amount (100 ng/mL) by employing a piezoresistive SU-8/CB microcantilever platform for the first time. In 2020, Dilip et al. [160] tested cardiac troponin-I by using a a SiN–PolySi–SiO_2_ composite microcantilever modified with HIgG and Anti-HIgG.

#### 4.3.2. Antibiotics

As the most important invention in medical history, antibiotics have become a double-edged sword. The superbacteria produced by the overuse and abuse of antibiotics seriously threaten human health. Therefore, antibiotics are important detection objects of genetic-probe-modified microcantilevers.

In 2008, Ndieyira et al. [161] successfully used a drug-sensitive mucopeptide analogue (DAla) as a sensitive layer of a microcantilever to detect vancomycin, and quantitatively analyzed the interactions in antibiotic–mucopeptide binding. In 2013, Hou et al. [162] successfully detected oxytetracycline by using a sensor array consisting of self-assembled monolayers (SAMs) of OTC-specific aptamers as a working cantilever sensitive layer and 6-mercapto-1-hexanol SAMs as a reference cantilever sensitive layer. In the following years, by using a similar method, the group successfully detected multiple antibiotics such as kanamycin [163], fumonisin B-1 [164], and nucleolin [165].

#### 4.3.3. Heavy Metal Ions

Heavy metals are metals with a density greater than 5 g/cm^3^. Most heavy metal elements are environmental pollutants, which seriously threaten human health. Therefore, heavy metal ions are also detection targets of microcantilevers. In 2004, Cherian et al. [166] functionalized a microcantilever with metal-binding protein AgNt84-6 that had the ability to bind multiple ions of Ni^2+^, Zn^2+^, Co^2+^, Cu^2+^, Cd^2+^, and Hg^2+^. This research demonstrated that a microcantilever can be used to discriminate multiple metal ions. In 2009, Xu et al. [167] grafted a Gly–Gly–His (GGH) tripeptide to the 3-mercaptopropionic acid (MPA) layer on the microcantilever gold surface. Then, they studied the interaction between tripeptide Gly–Gly–His and Cu^2+^ under different environmental conditions and analyzed the mechanism of microcantilever deflection. Since 2012, Peng has functionalized microcantilevers by multiple methods involving sensitive layers for detecting heavy metal ions, including benzo-9-crown-3 doped hydrogen for Be^2+^ [168], a specific Pb²⁺-dependent DNAzyme molecule for Pb²⁺ [169], and benzo-9-crown-3 polymer brush for Be^2+^ [170]. In these studies, the microcantilever sensor modified by DNAzyme not only exhibited high selectivity to Pb²⁺ (10^−^⁸ M), but could be regenerated by flowing through a strong Pb²⁺ chelator (1, 4, 7, 10-tetraazacyclododecane-1, 4, 7, 10-tetraacetic acid). In 2017, You et al. [171] reported a mass-amplified silver ion sensor (MAIS) based on a resonance microcantilever with cytosine-based DNA as a sensitive layer. To enhance the sensitivity, GNPs that do not affect the specific matching between silver ion and cytosine-DNA layer were introduced to improve resonance frequency shift. Furthermore, genetically modified cantilever sensors can also be used with other environmental pollutants such as okadaic acid (OA) [172] and in food security, such as with profenofous pesticide residues in vegetables [173] and the hepatic toxin microcystin–leucine–arginine (MC-LR) [174].

### 4.4. Integrated Detection

In early detection, the microcantilever is usually placed in the stage or testing pool with the samples, and then the deflection or vibration changes of the sensor are measured by an instrument such as an AFM or PSD. This detection process not only fails to show the advantages of microcantilever, including small volume, simple structure, and easy integration, but also limits the detection scenario to the laboratory, hindering the application and development of microcantilever sensors in rapid and portable detection. Micro total analysis systems (μTASs), proposed at the beginning of this century, have the characteristics of small size, high integration, and excellent compatibility. Combined with optics, electrochemistry, and other detection methods, the μTAS has become an indispensable analytical technology in the fields of biology, medicine, chemistry, and the environment. Therefore, the μTAS is an ideal choice for a microcantilever to avoid the constraints of the laboratory environment as well as to realize real-time use, miniaturization, and commercialization.

**Figure 14:**
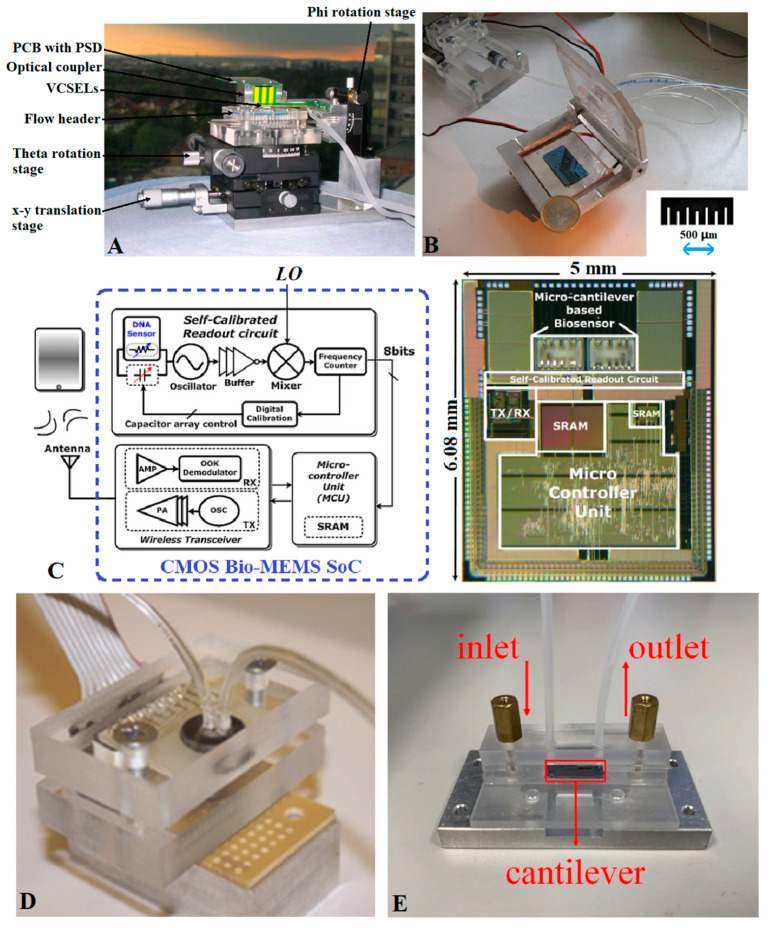
(**A**) Integrated testing equipment of Lechuga et al. [67]; (**B**) integrated testing equipment of Ricciardi et al. [175]; (**C**) integrated testing equipment of Huang et al. [176]; (**D**) integrated testing equipment of Khemthongcharoen et al. [142]; (**E**) integrated testing equipment of Wang et al. [184].

As shown in Figure 14A, in 2006, Lechuga et al. [67] integrated a silicon-based microcantilever array, polymer microfluidic chip, vertical-cavity surface-emitting laser, segmented photodetector, and other modules to prepare a small biochemical detection system with an Atomic Force Microscopy (AFM) detection limit. As shown in Figure 14B, in 2010, Ricciardi et al. [175] integrated a microcantilever array and piezoelectric driver into a μTAS chip. With the help of external optical instruments, the chip can complete *Salmonella* detection within 40 min. As shown in Figure 14C, in 2011, Huang et al. [176,177,178] integrated a microcantilever, self-calibrated readout circuit, programmable microcontroller, voltage regulator, wireless transceiver, and other modules on a single-chip SoC by adopting a 0.35 μm standard CMOS process, and then realized wireless detection of HBV by combining it with microfluidic technology. As shown in Figure 14D, in 2015, Khemthongcharoen et al. [142] prepared an integrated microfluidic detection chip with PCR technology and a pressure-sensitive microcantilever as the core. Compared with conventional PCR, the sensitivity of the integration system is 10 times higher. Starting in 2014, Wang et al., reported a series of works about microcantilever integration, including surface antibody modification [179,180], and the design and fabrication of a microcantilever with cavity, piezoelectric drive, and frequency-tracking circuit [181,182,183]. The latest research of this team, in 2021 [184], had a microcantilever prepared on an SOI substrate by standard CMOS processes such as RIE and PECVD. After modification by the antigen–antibody system, the microcantilever was integrated into a microfluidic chip, as shown in Figure 14E. The integrated detection system can detect alpha-fetoprotein under the amplification of nanoparticles.

## 5. Conclusions and Prospects

Since the microcantilever and genetic probe technologies were first combined in 2000, the new sensor has been used for cutting-edge applications such as the determination of nucleic acids (DNA or RNA) and its derivatives (oligonucleotides or polynucleotides), the exploration of complementary hybridization reaction mechanisms, and analysis of the gene transcription process. Nowadays, this technology has gradually been applied to conventional instruments closely related to daily life, such as virus, bacteria, and cell detection (HBV, HCV, HIV, HPV), analysis of common drugs (oxytetracycline, kanamycin, etc.), the detection of heavy metal ions (Pb^2+^, Ag^+^), and early warning of food pesticide residues (Profenofos and MC-LR). During the transformation process, probe immobilization and complementary hybridization are no longer only the core procedures for sensitive layer preparation to expand the application range of microcantilever sensors, but are also a means to reveal deflection mechanisms and perform the analysis of influencing factors such as DNA molecular density, chain length, and conformational entropy. Meanwhile, the detection signal extraction and processing of microcantilevers have changed from optical interference or optical deflection relying on an AFM system to electrical methods such as capacitance, piezoelectricity, and piezoresistivity, which are more suitable for detection instruments’ miniaturization, integration, and portability.

However, there are still many obstacles to the commercialization of microcantilever sensors modified by a genetic probe. First of all, although the integration level of the microcantilever sensor has improved greatly, and already includes driving, detecting, and signal processing, many of the biochemical analysis steps, such as the mixing, separation, and enrichment of samples, still need to be carried out in the laboratory before detection. These operations are not only time-consuming and laborious, but also require professional knowledge. The combination of genetic probe modification of a microcantilever sensor and μTAS can alleviate this problem, but the integrated detection system is still in the prototype exploration stage. Secondly, the deflection and sensitivity of a genetic-probe-modified microcantilever are determined by many factors, such as the molecular force between DNA strands, hydration force, configuration entropy, and osmotic pressure. However, most detection principles and models can only qualitatively or semiquantitatively explain sensor deflection in a specific detection process, without accurately predicting the deflection trend of a microcantilever sensor. Thirdly, the influence of temperature, salinity, humidity, pH, and other environmental factors on microcantilever sensor detection cannot be ignored, but current research still focuses on individual detection under specified environmental conditions, and there are still few comprehensive conclusions about various environmental factors. In order to solve the above problems, promoting the innovation of genetic-probe-modified microcantilever sensors and achieving the ultimate goal of integrated and portable detection, researchers not only need to establish a quantitative detection model including environmental factors by means of combining simulation analysis with detection test, but also must realize the cooperation of representatives of various fields such as device design, preparation technology, and application promotion.

## Data Availability

Not applicable.
